# Supplementary material for: Starch Synthesis-Related Genes (SSRG) Evolution in the Genus Oryza
Source: Plants (Basel). 2021 May 25;10(6):1057. doi: 10.3390/plants10061057 (PMC8229393; doi:10.3390/plants10061057)
Supplement: Supplementary file 1 [file plants-10-01057-s001.zip › MTable S1.pdf]

Table S1. Full information about each of the Starch Synthesis-Related Genes (SSRGs) analyzed.

Locus name	# of Transcripts	RAP-DB/Ensembl ID	Location	Start	End	Size (pb)	ID	*E-value*
AGPL1	1	*Os05g0580000*	Chr. 5	28871811	28877272	5462		
AGPL3	2	*Os03g0735000*	Chr. 3	30099369	30104572	5204		
AGPL4	1	*Os07g0243200*	Chr. 7	7983125	7989283	6159		
AGPS2a	4	*Os08g0345800*	Chr. 8	15666389	15672583	6248		
ALK	1	*Os06g0229800*	Chr. 6	6748398	6753302	4916		
DPEI	1	*Os07g0627000*	Chr. 7	25980423	25984853	4431		
GBSSII	2	*Os07g0412100*	Chr. 7	12916883	12924202	7320		
ISA	1	*Os08g0520900*	Chr. 8	25893657	25900576	6920		
PUL	1	*Os04g0164900*	Chr. 4	4408357	4418889	10533		
SBE1	4	*Os06g0726400*	Chr. 6	30897378	30905803	8426		
SBE3	1	*Os02g0528200*	Chr. 2	19355790	19367127	11338		
SSI	1	*Os06g0160700*	Chr. 6	3079296	3086808	7513		
SSII 1	1	*Os10t0437600*	Chr. 10	15673243	15681075	7833		
SSII 2	2	*Os02g0744700*	Chr. 2	31233292	31238210	4929		
SSIII 1	2	*Os04g0624600*	Chr. 4	31751600	31759420	7821		
SSIII 2	1	*Os08g0191433*	Chr. 8	5353697	5363276	9580		
SSIV 1	4	*Os01g0720600*	Chr. 1	30032428	30041425	8998		
SSIV 2	2	*Os05g0533600*	Chr. 5	26485770	26493983	8214		
Wx	2	*Os06g0133000*	Chr. 6	1765622	1770653	5032		
AGPL1	1	*BGIOSGA017490*	Chr. 5	30220701	30222552	3388	99.9	0.0
AGPL3	1	*BGIOSGA009855*	Chr. 3	34318562	34322802	4241	99.6	0.0
AGPL4	1	*BGIOSGA024540*	Chr. 7	7995391	7999961	4571	99.6	0.0
AGPS2a	1	*BGIOSGA027135*	Chr. 8	16750552	16746477	4076	99.5	0.0
ALK	1	*BGIOSGA022586*	Chr. 6	7778365	7782784	4420	99.9	0.0
DPEI	1	*BGIOSGA026185*	Chr. 7	24042478	24046564	4087	99.7	0.0
GBSSII	1	*BGIOSGA024424*	Chr. 7	12148263	12152181	3919	99.2	0.0
ISA	1	*BGIOSGA026650*	Chr. 8	27633755	27640589	6835	99.9	0.0
PUL	1	*BGIOSGA015875*	Chr. 4	3476296	3488775	12480	98.2	0.0
SBE1	1	*BGIOSGA020506*	Chr. 6	32596001	32603277	7277	99.8	0.0
SBE3	1	*BGIOSGA006344*	Chr. 2	20764778	20775734	10957	99.7	0.0
SSI	1	*BGIOSGA021860*	Chr. 6	3609454	3616267	6814	100.0	0.0
SSII 1	1	*BGIOSGA033011*	Chr. 10	14120504	14127623	7120	99.6	0.0
SSII 2	1	*BGIOSGA005631*	Chr. 2	33274118	33278322	4215	99.8	0.0
SSIII1	1	*BGIOSGA014316*	Chr. 4	30784815	30792005	7186	99.9	0.0
SSIII 2	1	*BGIOSGA028122*	Chr. 8	5614172	5624994	10823	99.7	0.0
SSIV 1	1	*BGIOSGA000900*	Chr. 1	33261071	33269378	8308	99.7	0.0
SSIV 2	1	*BGIOSGA020250*	Chr. 5	27869304	27876940	7637	99.7	0.0
Wx	1	*BGIOSGA022241*	Chr. 6	1931535	1935014	3480	100.0	0.0
AGPL1	1	*ORUFI05G29020*	Chr. 5	25558424	25561810	3387	100.0	0.0
AGPL3	1	*ORUFI03G34620*	Chr. 3	28543622	28547924	4303	99.8	0.0

AGLP4	1	*ORUFI07G08660*	Chr. 7	7250572	7256218	5647	99.9	0.0
AGPS2a	2	*ORUFI08G13010*	Chr. 8	13811400	13817275	5876	100.0	0.0
ALK	1	*ORUFI06G08580*	Chr. 6	6231488	6236538	5051	99.5	0.0
DPEI	2	*ORUFI07G24050*	Chr. 7	22751548	22756102	4555	100	0.0
GBSSII	1	*ORUFI07G11630*	Chr. 7	11317247	11324432	7186	99.1	0.0
ISA	1	*ORUFI08G23320*	Chr. 8	23528272	23535101	6830	99.9	0.0
PUL	1	*ORUFI04G02940*	Chr. 4	3519334	3532236	12903	98.3	0.0
SBE1	5	*ORUFI06G29940*	Chr. 6	27526184	27533602	7419	99.8	0.0
SBE3	1	*ORUFI02G19870*	Chr. 2	18185004	18195898	10895	100.0	0.0
SSI	1	*ORUFI06G04040*	Chr. 6	2791984	2798799	6816	99.9	0.0
SSII 1	3	*ORUFI10G11670*	Chr. 10	13668717	13679236	10520	99.7	0.0
SSII 2	2	*ORUFI02G33990*	Chr. 2	29295244	29299474	4231	99.8	0.0
SSIII 1	1	*ORUFI04G27570*	Chr. 4	27123710	27130905	7196	99.9	0.0
SSIII 2	3	*ORUFI08G05900*	Chr. 8	4762126	4775482	13357	99.4	0.0
SSIV 1	3	*ORUFI01G32260*	Chr. 1	27539416	27547732	8317	99.9	0.0
SSIV 2	2	*ORUFI05G25430*	Chr. 5	23281640	23290019	8380	99.8	0.0
Wx	2	*ORUFI06G02030*	Chr. 6	1559398	1564292	4895	99.9	0.0
AGPL1	2	*ONIVA05G29110*	Chr. 5	26996830	26999189	5239	100.0	0.0
AGPL3	1	*ONIVA03G34930*	Chr. 3	30015137	30019384	4248	99.6	0.0
AGPL4	1	*ONIVA07G07390*	Chr. 7	6096533	6102180	5648	99.7	0.0
AGPS2a	2	*ONIVA04G29180*	Chr. 4	27995462	28001390	5929	99.5	0.0
ALK	1	*ONIVA06G09520*	Chr. 6	6979112	6984349	5238	99.4	0.0
DPEI		**UNKNOWN**						
GBSSII	2	*ONIVA07G09560*	Chr. 7	9331482	9338655	7174	99.8	0.0
ISA	2	*ONIVA01G02600*	Chr. 1	1822103	1830976	8874	99.7	0.0
PUL	3	*ONIVA08G18600*	Chr. 8	20035949	20048691	12743	99.8	0.0
SBE1	6	*ONIVA06G30960*	Chr. 6	28851472	28859303	7832	99.8	0.0
SBE3	1	*ONIVA02G20920*	Chr. 2	19160312	19171267	10956	99.8	0.0
SSI	1	*ONIVA06G20230*	Chr. 6	18799660	18806464	6805	99.4	0.0
SSII 1	3	*ONIVA10G10320*	Chr. 10	11413747	11424234	10488	99.7	0.0
SSII 2	2	*ONIVA06G04720*	Chr. 6	3161289	3166670	5391	99.5	0.0
SSIII 1	2	*ONIVA04G24880*	Chr. 4	24741542	24749237	7696	99.9	0.0
SSIII 2	2	*ONIVA08G05260*	Chr. 8	4445515	4458770	13256	99.7	0.0
SSIV 1	3	*ONIVA01G33370*	Chr. 1	29128063	29136375	8313	99.9	0.0
SSIV 2	1	*ONIVA05G24550*	Chr. 5	24014997	24023064	8040	99.7	0.0
Wx	1	*ONIVA06G02500*	Chr. 6	1715144	1719189	4046	99.9	0.0
AGPL1	1	*OGLUM05G28600*	Chr. 5	29490471	29492029	3389	99.7	0.0
AGPL3	1	*OGLUM03G32900*	Chr. 3	31084033	31088270	4238	99.6	0.0
AGPL4	1	*OGLUM07G07800*	Chr. 7	7504668	7510317	5650	99.6	0.0
AGPS2a	1	*OGLUM08G12560*	Chr. 8	13673152	13677206	4055	99.8	0.0
ALK	1	*OGLUM06G08830*	Chr. 6	6894230	6899271	5050	99.5	0.0
DPEI	2	*OGLUM07G22950*	Chr. 7	24895289	24899871	4583	99.7	0.0
GBSSII	2	*OGLUM07G10960*	Chr. 7	12170159	12177340	7182	99.7	0.0

ISA	1	*OGLUM08G22090*	Chr. 8	24554177	24560987	6811	99.9	0.0
PUL	3	*OGLUM04G01860*	Chr. 4	2886872	2900509	13638	98.5	0.0
SBE1	7	*OGLUM06G29370*	Chr. 6	31128403	31137352	8950	99.8	0.0
SBE3	1	*OGLUM02G19180*	Chr. 2	20189625	20200539	10915	99.7	0.0
SSI	1	*OGLUM06G04130*	Chr. 6	2892170	2898982	6821	99.1	0.0
SSII 1	2	*OGLUM10G10900*	Chr. 10	14772075	14780343	8269	97.4	0.0
SSII 2	2	*OGLUM02G32940*	Chr. 2	32627141	32631778	4638	99.5	0.0
SSIII 1	3	*OGLUM04G25800*	Chr. 4	28951767	28958958	7192	98.0	0.0
SSIII 2	2	*OGLUM08G05570*	Chr. 8	4670325	4683755	13441	99.4	0.0
SSIV 1	2	*OGLUM01G33230*	Chr. 1	32640551	32648862	8312	99.5	0.0
SSIV 2	2	*OGLUM05G25180*	Chr. 5	26994912	27002552	7641	98.2	0.0
Wx	2	*OGLUM06G02020*	Chr. 6	1469264	1474035	4772	99.9	0.0
AGPL1	1	*ORGLA05G0234700*	Chr. 5	22419555	22421766	3384	98.9	0.0
AGPL3	1	*ORGLA03G0301700*	Chr. 3	27225984	27230182	4199	99.7	0.0
AGPL4	1	*ORGLA07G0073000*	Chr. 7	6872607	6877182	4516	99.8	0.0
AGPS2a	1	*ORGLA08G0104300*	Chr. 8	11519446	11523524	5891	99.5	0.0
ALK	1	*ORGLA06G0078600*	Chr. 6	6002051	6006473	4423	99.6	0.0
DPEI	1	*ORGLA07G0179000*	Chr. 7	18641028	18645164	4137	98.2	0.0
GBSSII	1	*ORGLA07G0097800*	Chr. 7	10557492	10561434	3943	99.5	0.0
ISA	1	*ORGLA08G0184300*	Chr. 8	19624422	19631228	6807	99.7	0.0
PUL	1	*ORGLA04G0016300*	Chr. 4	2098830	2109127	10308	99.6	0.0
	1	*ORGLA06G0243400*	Chr. 6	23629078	23642103	11836	99.4	0.0
SBE1	1	*ORGLA06G0237000*	Chr. 6	23121600	23127808	6209	99.6	0.0
SBE3	1	*ORGLA02G0162800*	Chr. 2	15767001	15777902	10902	99.8	0.0
SSI	1	*ORGLA06G0037300*	Chr. 6	2672463	2679274	6812	99.5	0.0
SSII 1	1	*ORGLA10G0098400*	Chr. 10	12560982	12568108	7127	99.8	0.0
SSII 2	1	*ORGLA02G0278400*	Chr. 2	25119935	25124140	4206	99.5	0.0
SSIII 1	1	*ORGLA04G0229100*	Chr. 4	23494796	23501988	7193	99.7	0.0
SSIII 2	1	*ORGLA08G0045500*	Chr. 8	3902326	3913414	11089	99.7	0.0
SSIV 1	1	*ORGLA01G0239700*	Chr. 1	21752449	21760765	8317	99.6	0.0
SSIV 2		**UNKNOWN**						
Wx	1	*ORGLA06G0020500*	Chr. 6	1582446	1585771	3326	99.6	0.0
AGPL1	1	*OBART05G27050*	Chr. 5	23324094	23326305	3384	99.8	0.0
AGPL3	1	*OBART03G33230*	Chr. 3	27626346	27630535	4190	99.7	0.0
AGPL4	1	*OBART07G08590*	Chr. 7	7013571	7017866	4296	99.8	0.0
AGPS2a	2	*OBART08G11760*	Chr. 8	11861898	11867788	5891	99.3	0.0
ALK	1	*OBART06G08250*	Chr. 6	6050736	6055714	4979	99.6	0.0
DPEI	2	*OBART07G22910*	Chr. 7	20754611	20758640	4030	98.2	0.0
GBSSII	2	*OBART07G11410*	Chr. 7	10578751	10585992	7142	99.5	0.0
ISA	1	*OBART08G20830*	Chr. 8	20404034	20410832	6699	99.7	0.0
PUL	1	*OBART04G02210*	Chr. 4	2180683	2193763	13081	99.6	0.0
SBE1	2	*OBART06G27990*	Chr. 6	25397160	25403370	6211	99.6	0.0
SBE3	1	*OBART02G18870*	Chr. 2	17066878	17077468	10591	99.7	0.0

SSI	1		*OBART06G04010*	Chr. 6	2754467	2761274	6808	99.4	0.0
SSII 1	2		*OBART10G11180*	Chr. 10	12596913	12604730	7818	99.8	0.0
SSII 2	1		*OBART02G32300*	Chr. 2	27493261	27497466	4206	99.5	0.0
SSIII 1		1	*OBART04G26440*	Chr. 4	24614479	24621672	7194	99.6	0.0
SSIII 2	3		*OBART08G05320*	Chr. 8	4222533	4234438	11906	99.6	0.0
SSIV 1	2		*OBART01G29190*	Chr. 1	25014283	25022599	8317	99.6	0.0
SSIV 2	4		*OBART05G23810*	Chr. 5	21216867	21225276	8310	99.5	0.0
Wx	1		*OBART06G02000*	Chr. 6	1521439	1525491	4094	99.6	0.0
AGPL1	4		*OMERI05G23130*	Chr. 5	25687807	25689281	7452	98.6	0.0
AGPL3	3		*OMERI03G29830*	Chr. 3	31404907	31420237	15331	98.7	0.0
AGPL4	1		*OMERI07G06990*	Chr. 7	7300131	7308247	8117	99.8	0.0
AGPS2a	1		*OMERI09G03640*	Chr. 9	5815979	5826226	10248	87.4	4.3E-71
ALK	1		*OMERI06G09850*	Chr. 6	8591311	8596588	5278	98.6	0.0
DPEI	1		*OMERI07G19310*	Chr. 7	22508514	22522342	13829	99.0	0.0
GBSSII			**UNKNOWN**						
ISA	1		*OMERI02G01830*	Chr. 2	1481268	1491073	9806	99.6	0.0
PUL	3		*OMERI04G02160*	Chr. 4	3050487	3064210	13418	98.5	0.0
SBE1	6		*OMERI06G27930*	Chr. 6	31435504	31444602	9099	99.4	0.0
SBE3	1		*OMERI02G18870*	Chr. 2	20742387	20758950	16564	99.0	0.0
SSI	1		*OMERI06G04610*	Chr. 6	3672873	3680742	7870	97.1	0.0
SSII 1	2		*OMERI04G07060*	Chr. 4	11369715	11377827	8113	98.7	0.0
SSII 2	1		*OMERI06G09850*	Chr. 6	8591311	8596588	5278	88.7	1.3E-142
SSIII 1	1		*OMERI04G21470*	Chr. 4	26281268	26296875	15151	97.9	0.0
	1		*OMERI04G21490*	Chr. 4	26300044	26307350	7307	97.9	0.0
SSIII 2		1	*OMERI08G05080*	Chr. 8	4840603	4852759	12157	99.5	0.0
SSIV 1	1		*OMERI01G26630*	Chr. 1	27952587	27955762	3176	97.1	0.0
		4	*OMERI01G26980*	Chr. 1	28230264	28240799	10536	97.1	0.0
SSIV 2	1		*OMERI05G21310*	Chr. 5	24160650	24175895	14194	99.6	0.0
Wx	3		*OMERI06G01920*	Chr. 6	1676732	1682574	5235	97.9	0.0
AGPL1	1		*OPUNC05G24670*	Chr. 5	30120865	30121813	3375	96.7	0.0
AGPL3	1		*OPUNC03G30430*	Chr. 3	32831814	32836265	4452	95.3	0.0
AGLP4	1		*OPUNC07G08120*	Chr. 7	8462242	8467841	5600	94.3	0.0
AGPS2a	2		*OPUNC08G10720*	Chr. 8	15575237	15580527	5291	97.6	0.0
ALK	1		*OPUNC06G08000*	Chr. 6	6492378	6497173	4796	96.9	0.0
DPEI	1		*OPUNC07G21530*	Chr. 7	27721637	27726637	5001	89.0	2.0E-135
GBSSII	1		*OPUNC07G10360*	Chr. 7	13869752	13873662	3911	96.1	0.0
ISA	1		*OPUNC08G18890*	Chr. 8	27058634	27065585	6952	95.9	0.0
PUL		1	*OPUNC04G02180*	Chr. 4	3369443	3383422	13980	95.6	0.0
SBE1	4		*OPUNC06G25520*	Chr. 6	34223277	34229751	6475	97.5	0.0
SBE3	2		*OPUNC02G16910*	Chr. 2	21616213	21627157	10945	94.1	0.0
SSI	2		*OPUNC06G03820*	Chr. 6	2602107	2608648	6542	97.9	0.0
SSII 1	3		*OPUNC10G09440*	Chr. 10	18391785	18399027	7243	96.2	0.0
SSII 2	1		*OPUNC02G29850*	Chr. 2	34702141	34710291	8151	98.5	0.0

SSIII 1	1		*OPUNC04G23440*	Chr. 4	30084234	30092699	8466	95.1	0.0
SSIII 2	1		*OPUNC08G05170*	Chr. 8	4642721	4654350	11630	97.1	0.0
SSIV 1	1		*OPUNC01G29020*	Chr. 1	32623724	32631889	8166	94.4	0.0
SSIV 2	1		*OPUNC05G21230*	Chr. 5	27633570	27640939	7370	96.8	0.0
Wx	1		*OPUNC06G01860*	Chr. 6	1254773	1258390	3618	96.1	5.4E-170
AGPL1	1		*OB05G34620*	Chr. 5	19404719	19405022	3789	93.4	2.1E-122
AGPL3	1		*OB03G40320*	Chr. 3	24403484	24407693	4210	89.1	1.1E-74
AGPL4	1		*OB07G16320*	Chr. 7	4817190	4823741	6552	94.0	1.3E-71
AGPS2a	1		*OB08G20190*	Chr. 8	9379642	9384572	6348	92.4	1.6E-126
ALK	1		*OB06G17800*	Chr. 6	5218731	5224167	5437	94.2	0.0
DPEI			**UNKNOWN**						
GBSSII	1		*OB0037G10230*	Chr. 7			4776	90.5	2.9E-159
ISA	1		*OB08G27630*	Chr. 8	16176164	16184129	7966	89.5	1.2E-93
PUL		1	*OB04G11820*	Chr. 4	1712825	1725559	12735	90.1	3.1E-98
SBE1		1	*OB06G35740*	Chr. 6	21520777	21526573	5797	94.4	0.0
SBE3	1		*OB02G26660*	Chr. 2	13948443	13958806	10364	88.7	1.6E-78
SSI	1		*OB06G13760*	Chr. 6	2200850	2208525	7462	93.7	0.0
SSII 1	1		*OB10G18500*	Chr. 10	8851531	8863293	8050	93.6	0.0
SSII 2	1		*OB02G39280*	Chr. 2	23179404	23191306	11218	95.1	0.0
SSIII 1	1		*OB04G32800*	Chr. 4	18435317	18443066	7750	91.6	0.0
SSIII 2	1		*OB08G15430*	Chr. 8	3676339	3688697	12359	92.7	0.0
SSIV 1	1		*OB01G38130*	Chr. 1	22754355	22763315	8961	90.3	0.0
SSIV 2	1		*OB05G31380*	Chr. 5	17372953	17379376	6424	92.5	0.0
Wx	1		*OB06G11980*	Chr. 6	1175992	1178897	2915	94.9	1.1E-108
AGPL1	1		*KN538814.1_FG015*	Chr. 5	24862240	24865615	3376	99.4	0.0
AGPL3	1		*KN539195.1_FG019*	Chr. 3	37651614	37655808	6268	99.2	0.0
AGPL4	1		*KN538938.1_FG006*	Chr. 3	24152577	24156527	4281	99.1	0.0
AGPS2a	1		*KN542860.1_FG001*	Chr. 8	12526401	12529162	5564	99.3	0.0
ALK	1		*KN538722.1_FG043*	Chr. 6	8195027	8198855	3964	99.6	0.0
DPEI	1		*KN538920.1_FG009*	Chr. 7	22602297	22605871	3575	98.2	0.0
GBSSII	1		*KN539544.1_FG005*	Chr. 7	12091388	12095326	3939	99.0	0.0
ISA	1		*KN538852.1_FG008*	Chr. 8	21633299	21637060	6624	98.4	0.0
PUL	1		*KN541174.1_FG002*	Chr. 4	1902073	1904954	8157	99.0	0.0
SBE1	1		*KN538785.1_FG052*	Chr. 6	30095026	30100130	6120	99.5	0.0
SBE3	1		*KN539270.1_FG007*	Chr. 2	18668529	18679912	10831	99.3	0.0
SSI	1		*KN538819.1_FG032*	Chr. 6	4298110	4304233	6798	99.4	0.0
SSII 1	1		*KN538870.1_FG026*	Chr. 10	11500196	11503685	9557	97.8	0.0
SSII 2	1		*KN539373.1_FG011*	Chr. 2	31221350	31225771	11863	99.2	0.0
SSIII 1	1		*KN539225.1_FG011*	Chr. 4	28535423	28542610	7198	98.0	0.0
SSIII 2	1		*KN539976.1_FG005*	Chr. 8	4385551	4396060	10509	99.3	0.0
SSIV 1	1		*KN538890.1_FG010*	Chr. 1	25879105	25889274	11120	97.3	0.0
SSIV 2	1		*KN542368.1_FG001*	Chr. 9	3065269	3071676	8426	98.6	0.0
Wx	1		*KN539033.1_FG002*	Chr. 6	2346028	2348767	3075	99.7	0.0

AGPL1	1		*LPERR05G22710*	Chr. 5	19941516	19941853	3365	90.2	1.2E-111
AGPL3	3		*LPERR03G27770*	Chr. 3	24280936	24285189	4254	94.8	1.9E-79
AGPL4	1		*LPERR07G07430*	Chr. 7	6441935	6453460	11426	93.8	8.7E-76
AGPS2a	2		*LPERR08G09730*	Chr. 8	9674267	9674615	5439	94.6	1.4E-151
ALK	1		*LPERR06G07610*	Chr. 6	5565368	5569799	4432	92.1	0.0
DPEI	1		*LPERR07G19870*	Chr. 7	19361114	19361592	4173	93.5	0.0
GBSSII	1		*LPERR05G07400*	Chr. 5	6809691	6818650	8960	91.0	9.7E-107
ISA	2		*LPERR08G17590*	Chr. 8	17935360	17943243	7884	89.6	2.1E-98
PUL		5	*LPERR04G01450*	Chr. 4	1637441	1651731	14299	92.5	2.4E-151
SBE1	1		*LPERR06G23440*	Chr. 6	20752621	20758851	6231	94.6	0.0
SBE3	1		*LPERR02G15110*	Chr. 2	13299407	13312765	13359	91.3	3.8E-144
SSI	1		*LPERR06G03680*	Chr. 6	2514855	2524553	9699	93.2	8.9E-98
SSII 1	3		*LPERR10G07960*	Chr. 10	9684239	9694403	10165	95.9	0.0
SSII 2	1		*LPERR02G26450*	Chr. 2	22543587	22548109	4523	94.0	0.0
	1		*LPERR06G07610*	Chr. 6	5565368	5569799	4432	89.1	5.4E-166
SSIII 1	1		*LPERR04G21480*	Chr. 4	20045750	20053049	7300	90.6	0.0
SSIII 2	1		*LPERR08G05100*	Chr. 8	4147149	4157275	10127	91.3	0.0
SSIV 1	5		*LPERR01G25000*	Chr. 1	21819891	21835575	15685	89.9	0.0
SSIV 2	2		*LPERR05G19650*	Chr. 5	17931037	17938653	7610	91.7	0.0
Wx	2		*LPERR06G01820*	Chr. 6	1223031	1227394	4364	92.5	8.9e-94
		1	*LPERR06G01810*	Chr. 7	1218230	1221309	3080	92.0	1.3E-89
