# Supplementary figures and images for: Starch Synthesis-Related Genes (SSRG) Evolution in the Genus Oryza
Source: Plants (Basel). 2021 May 25;10(6):1057. doi: 10.3390/plants10061057 (PMC8229393; doi:10.3390/plants10061057)

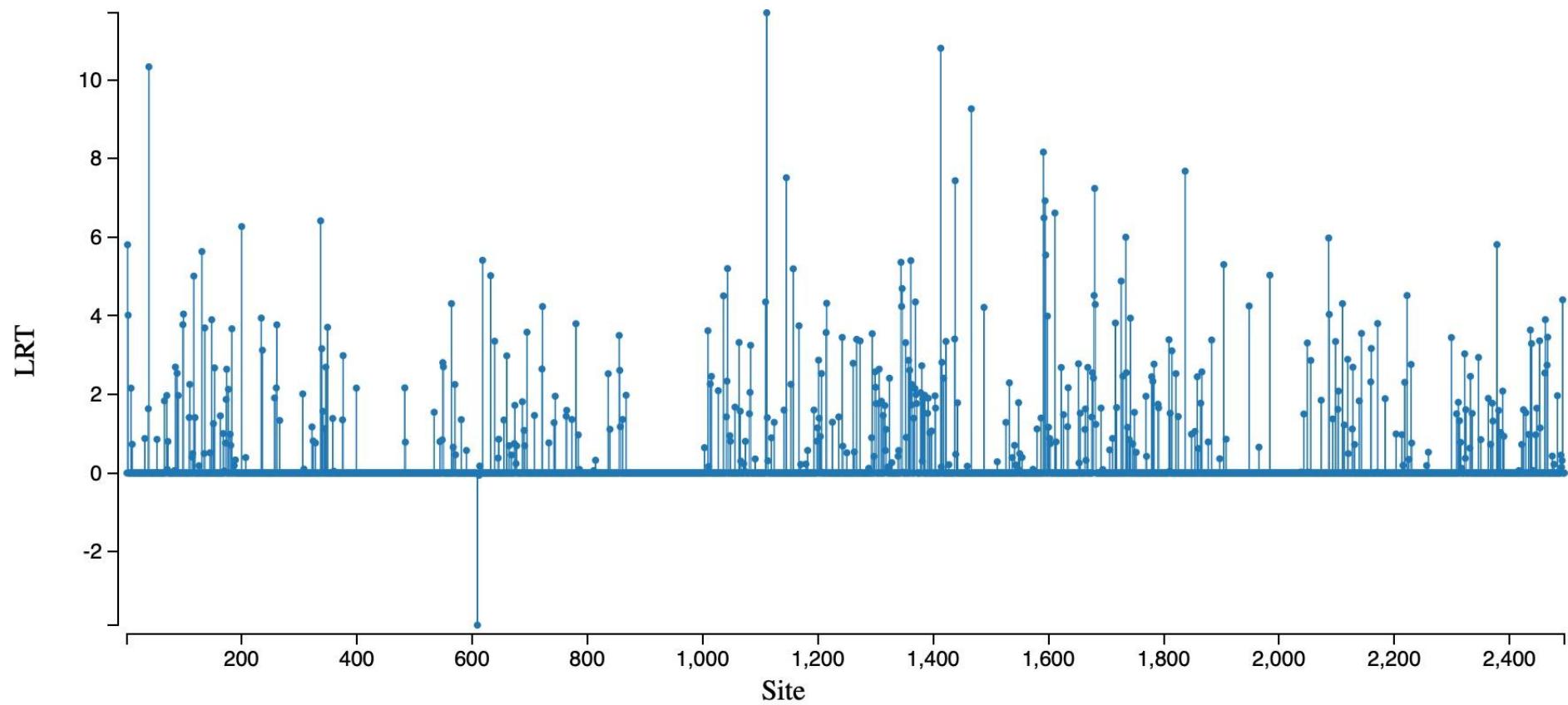

Supplement: Supplementary file 1 [file plants-10-01057-s001.zip › Figure S1.pdf]

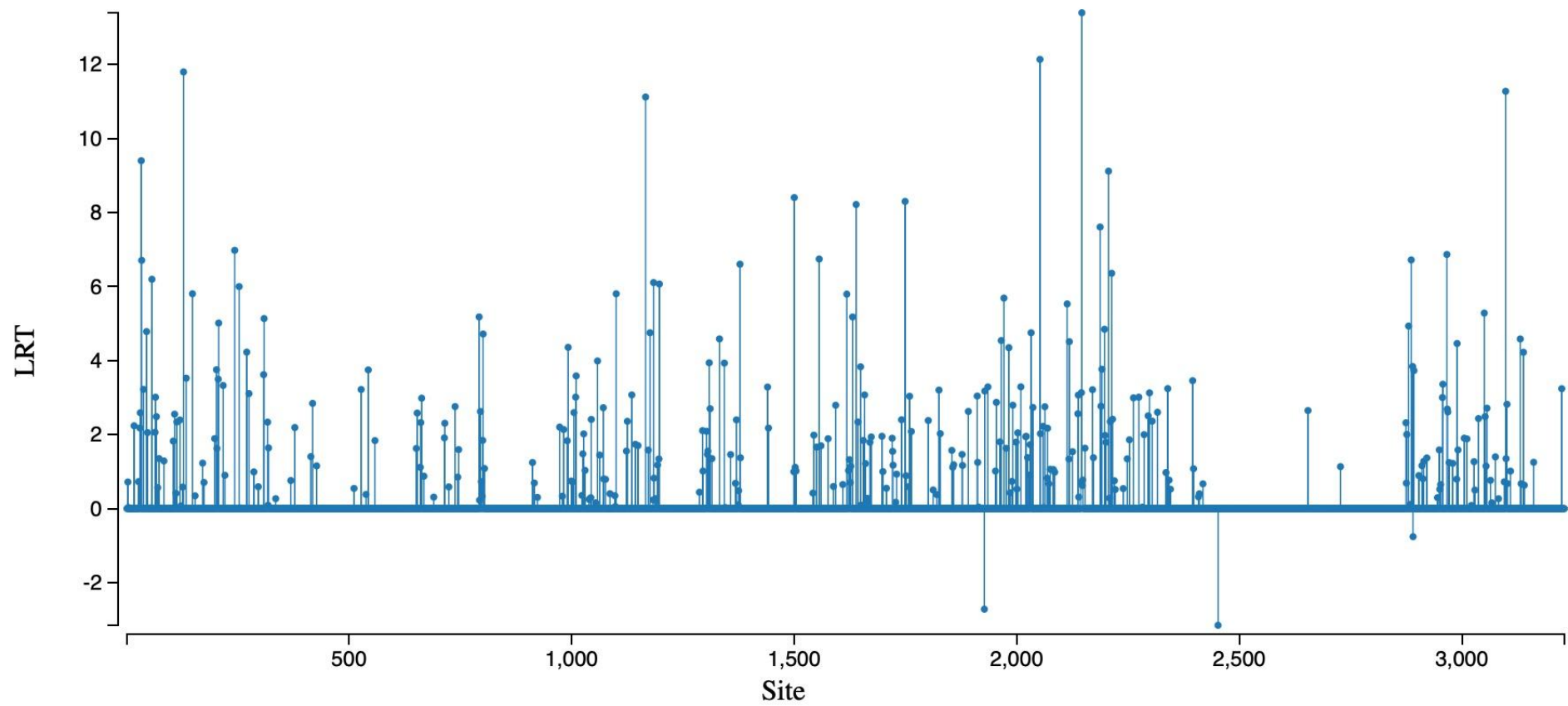

Supplement: Supplementary file 1 [file plants-10-01057-s001.zip › Figure S10.pdf]

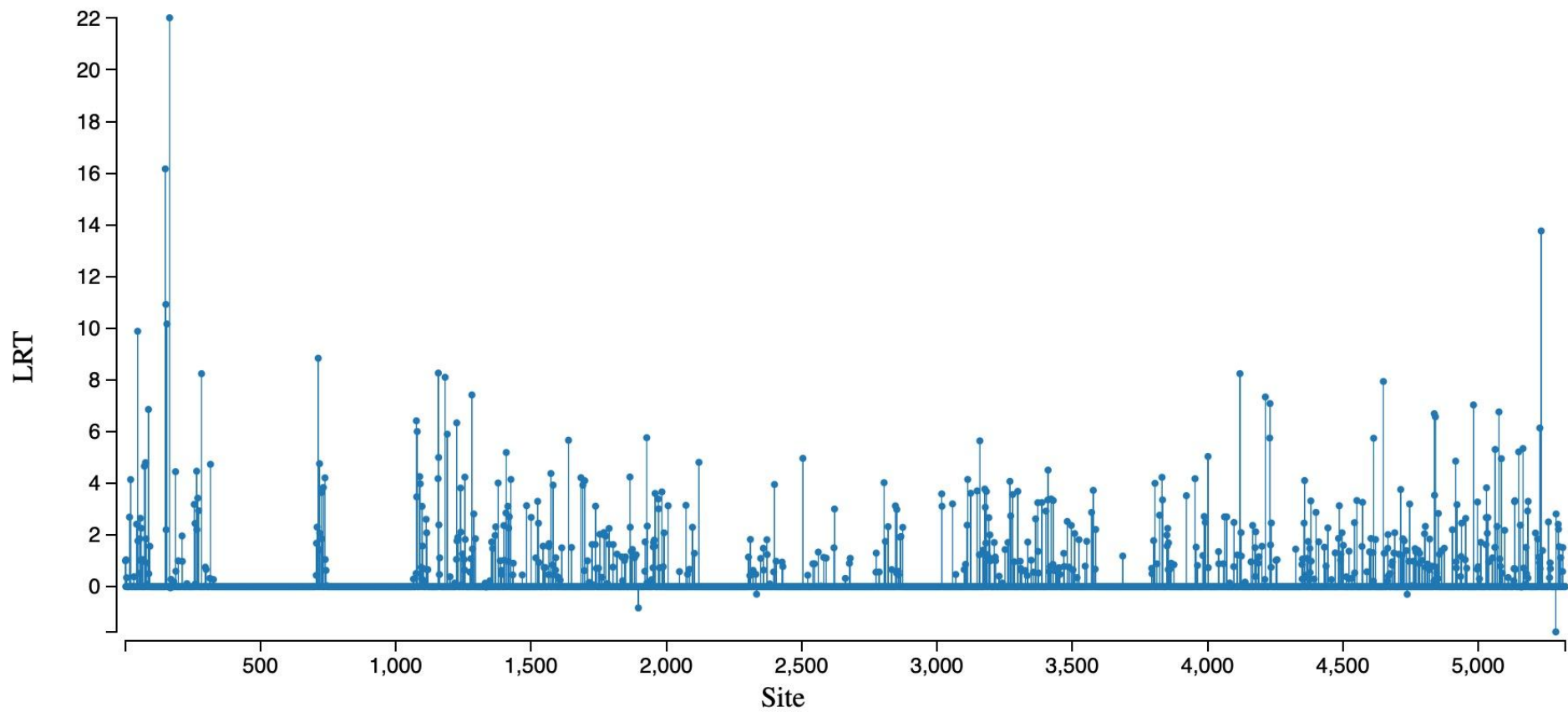

Supplement: Supplementary file 1 [file plants-10-01057-s001.zip › Figure S11.pdf]

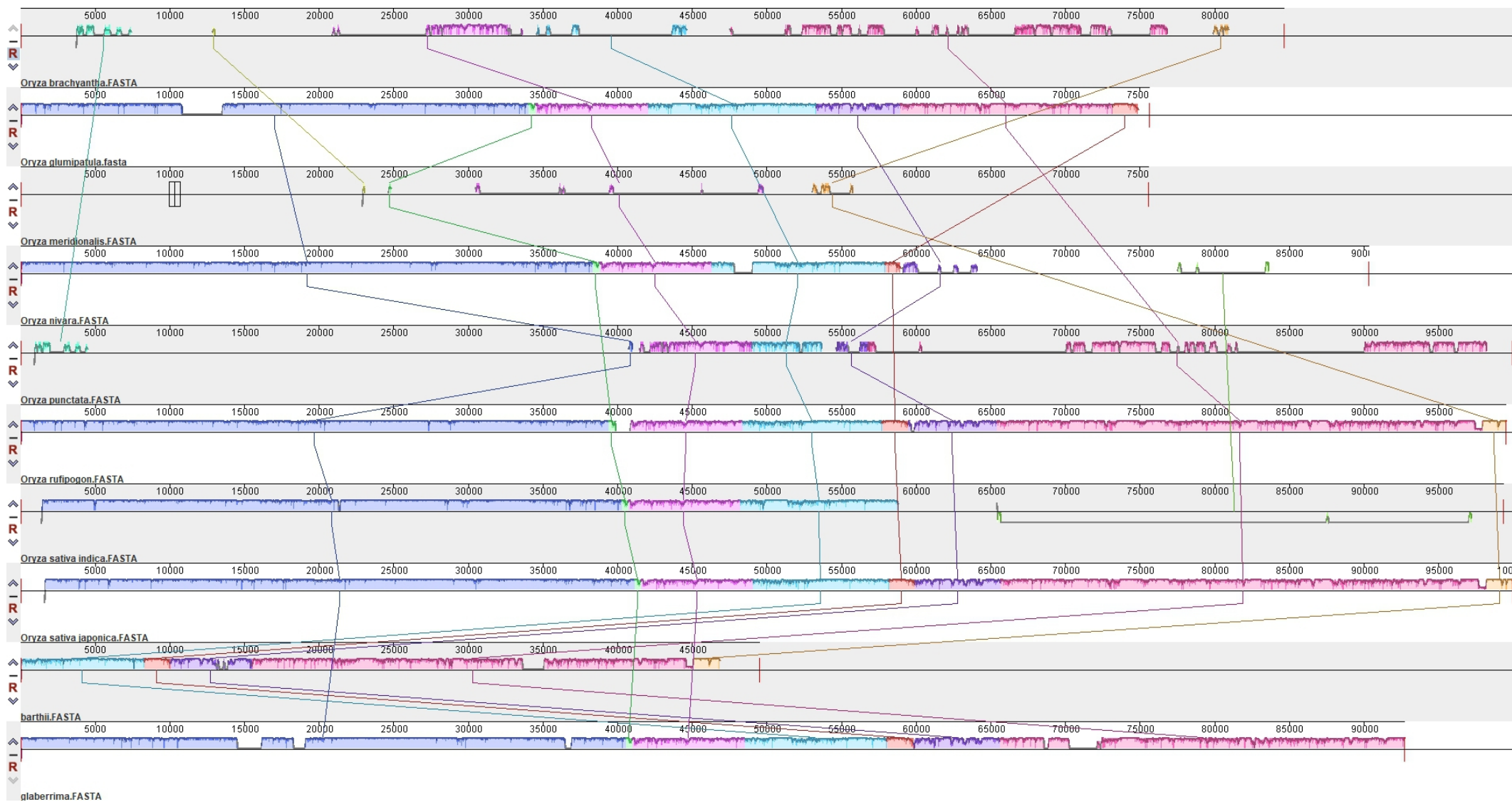

Supplement: Supplementary file 1 [file plants-10-01057-s001.zip › Figure S2.pdf]

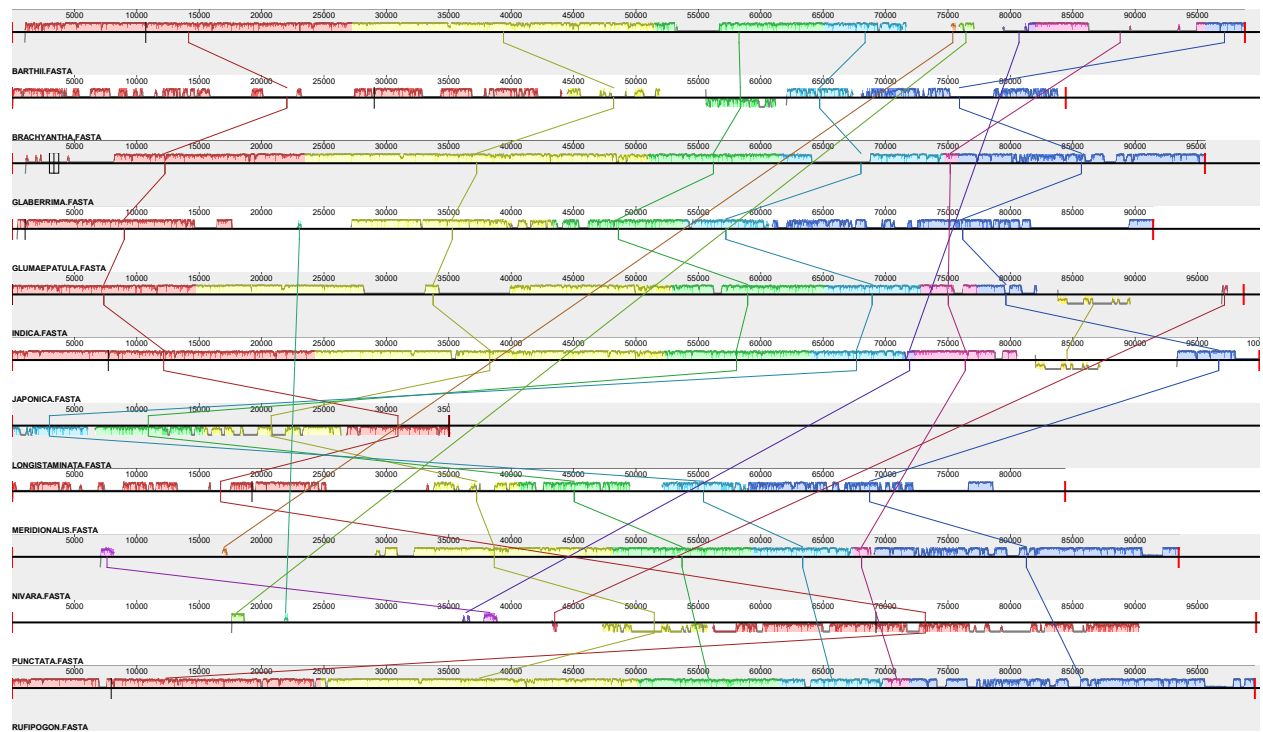

Supplement: Supplementary file 1 [file plants-10-01057-s001.zip › Figure S3.pdf]

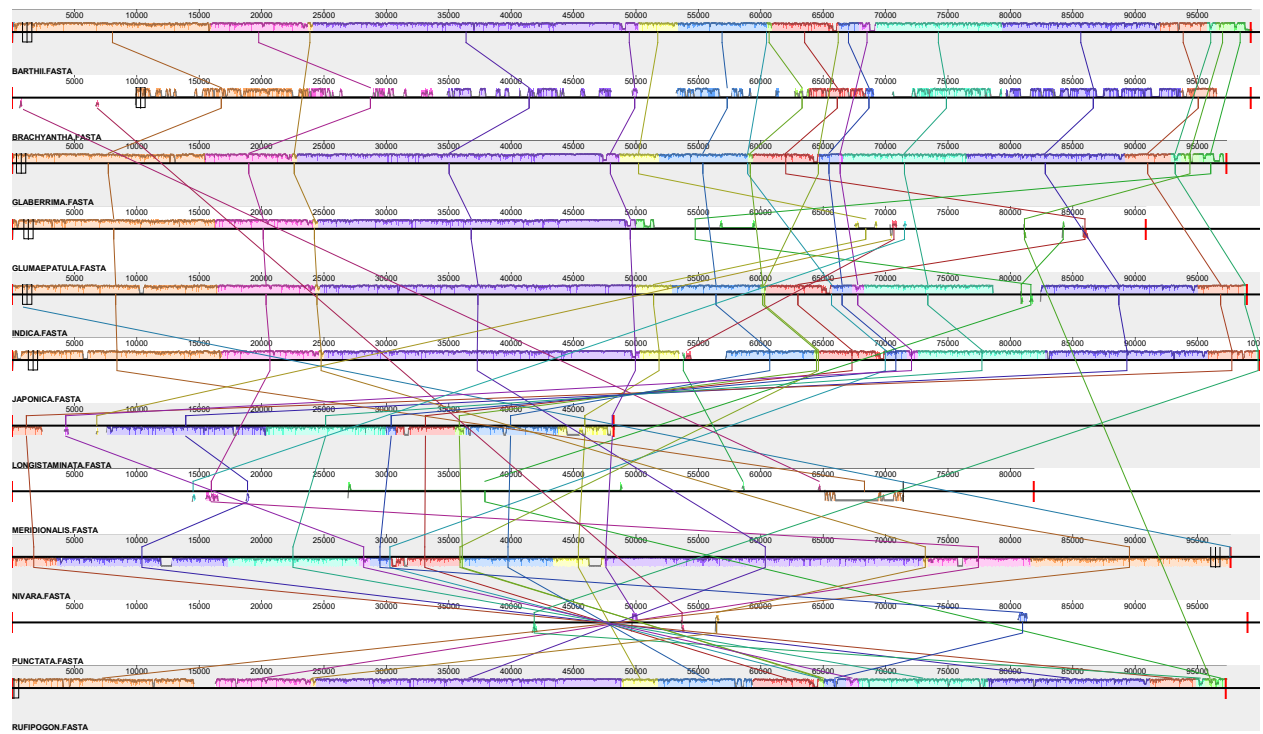

Supplement: Supplementary file 1 [file plants-10-01057-s001.zip › Figure S4.pdf]

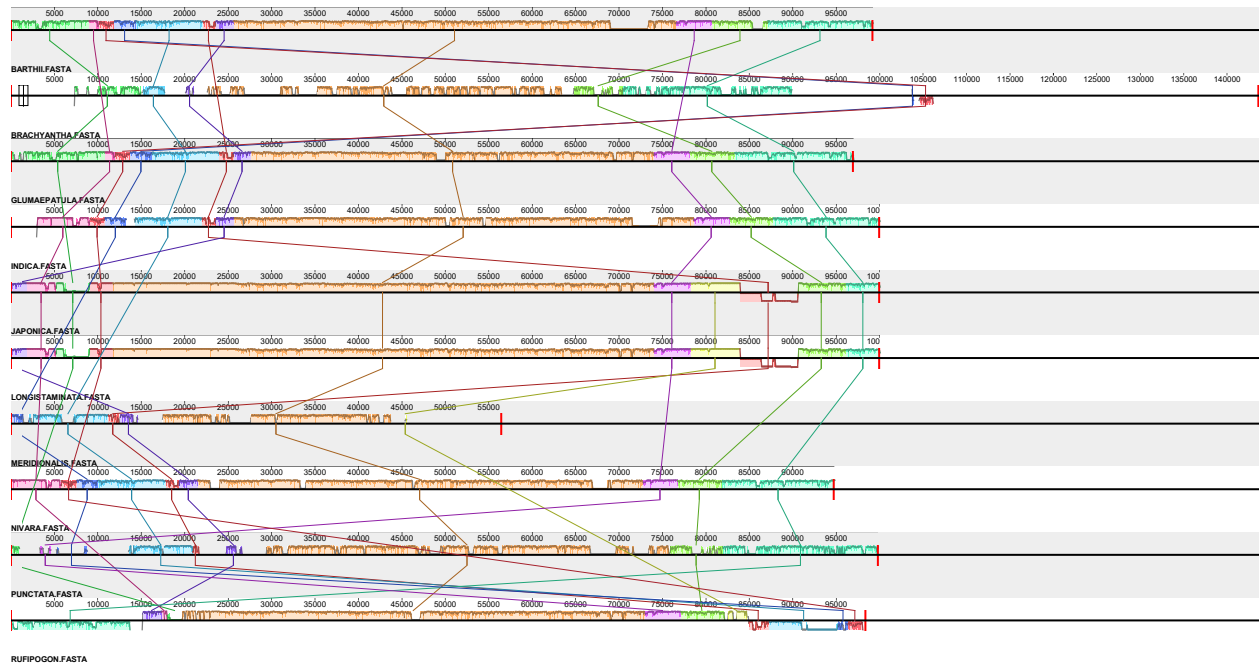

Supplement: Supplementary file 1 [file plants-10-01057-s001.zip › Figure S5.pdf]

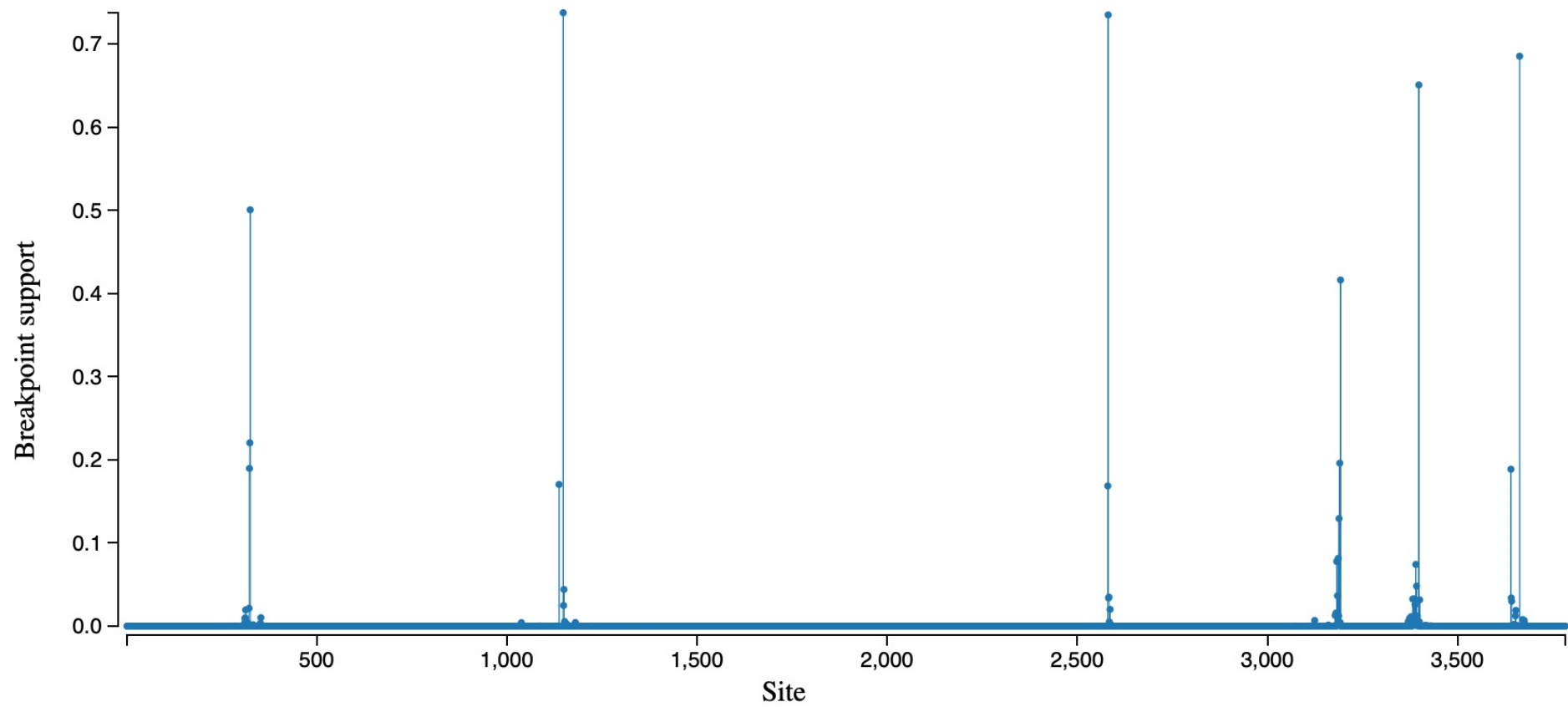

Supplement: Supplementary file 1 [file plants-10-01057-s001.zip › Figure S6.pdf]

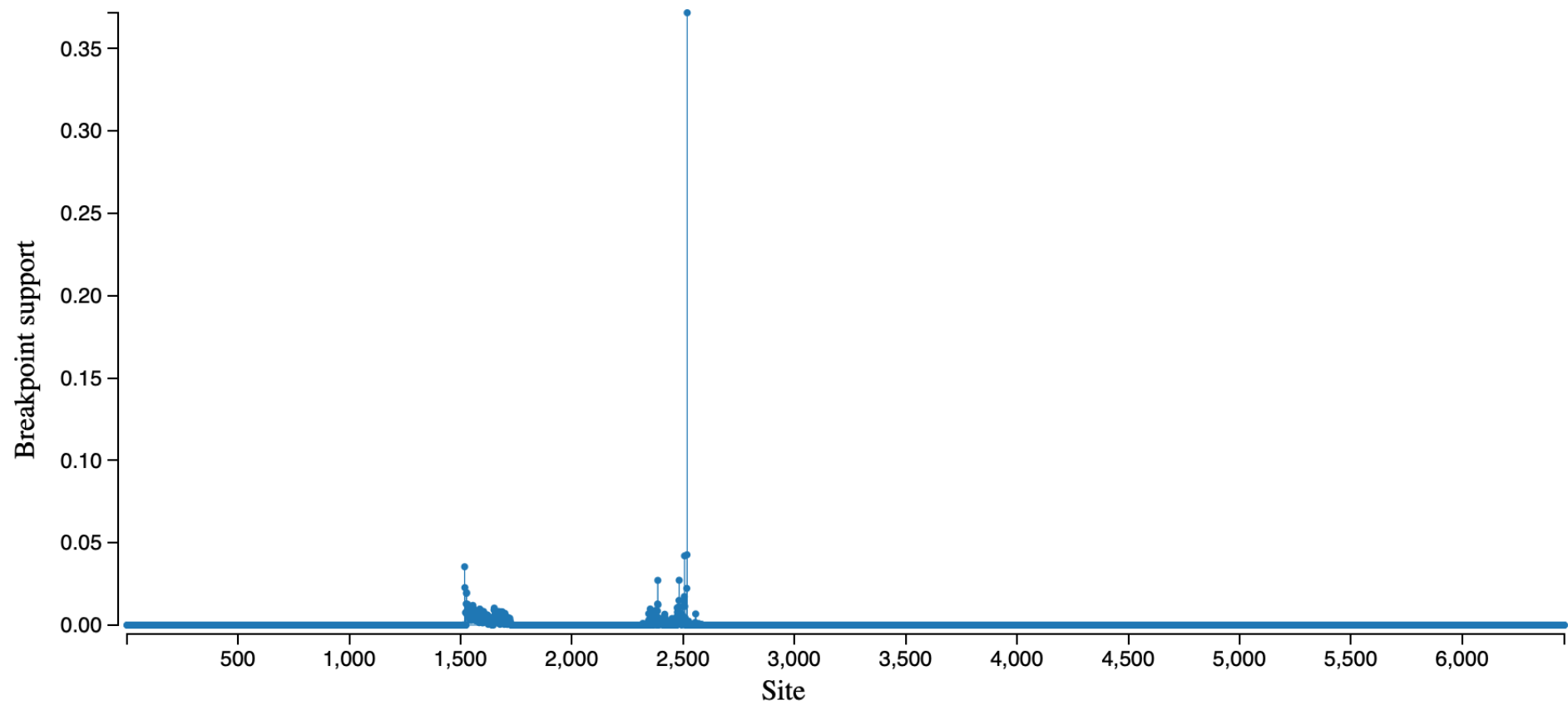

Supplement: Supplementary file 1 [file plants-10-01057-s001.zip › Figure S7.pdf]

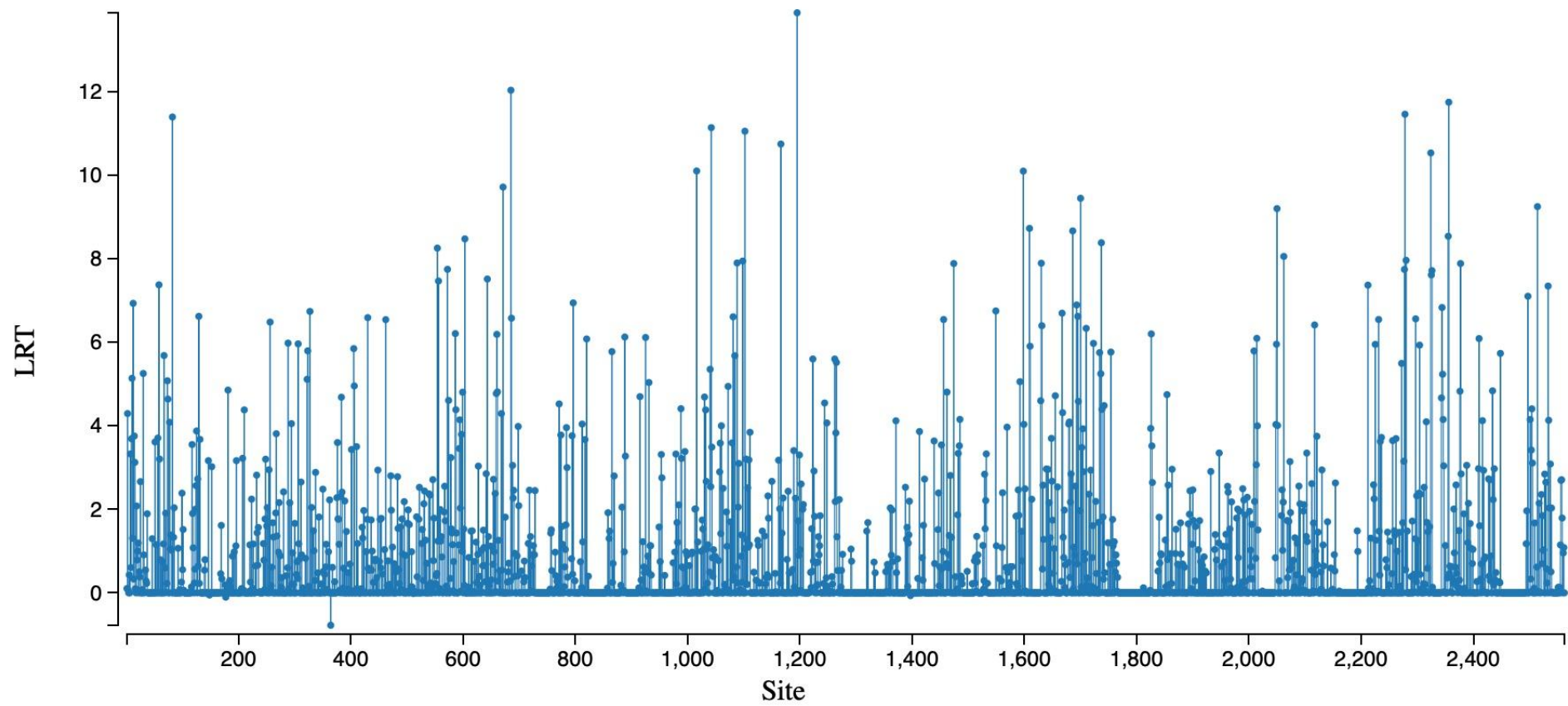

Supplement: Supplementary file 1 [file plants-10-01057-s001.zip › Figure S8.pdf]

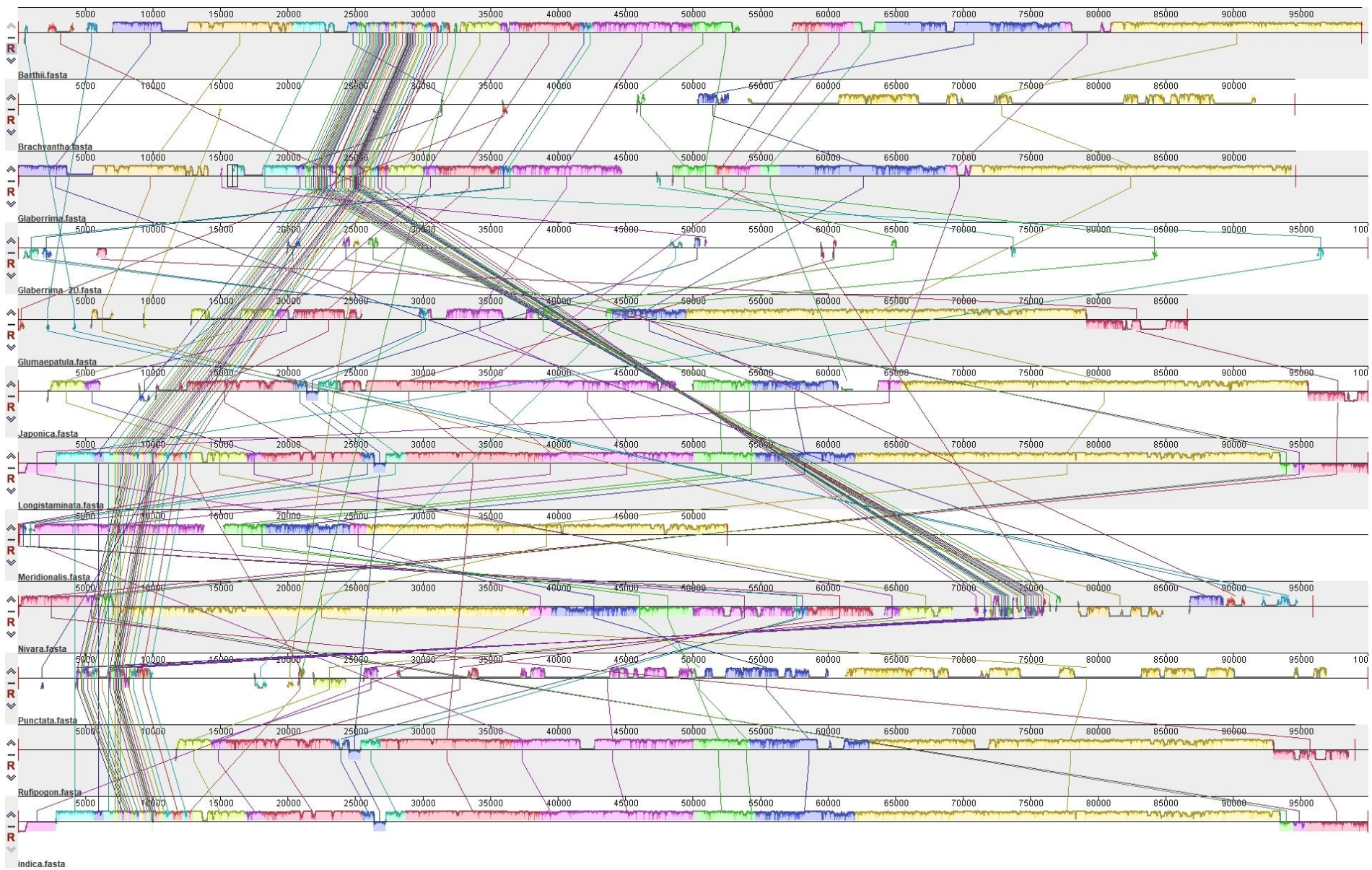

Supplement: Supplementary file 1 [file plants-10-01057-s001.zip › Figure S9.pdf]
